# Internet and Video Games: Causes of Behavioral Disorders in Children and Teenagers

**DOI:** 10.3390/children10010086

**Published:** 2022-12-31

**Authors:** Virginia Lérida-Ayala, José Manuel Aguilar-Parra, Rocío Collado-Soler, Marina Alférez-Pastor, Juan Miguel Fernández-Campoy, Antonio Luque-de la Rosa

**Affiliations:** Department of Psychology, University of Almería, 04120 Almería, Spain

**Keywords:** video games, behavioral disorders, technology, addiction, sleep disorders

## Abstract

Even though video games have been present among children for many years, children are using them more continuously and in an abusive and indiscriminate way nowadays because of the “technological boom”. It is affecting the behavior of children and adolescents. This is the reason why we are carrying out this systematic review. The main objective of this article is to investigate literature that directly connects the continuous and undifferentiated use of video games with the emergence of behavioral disorders in children and young people. The PRISMA statement was followed in the process of this article. We used SCOPUS, Web of Science and PubMed as databases, moreover, we searched studies with a scoping review. The results indisputably supported six out of seven of our hypotheses. We find that the excessive use of video games causes addiction to technology, aggressive behaviors, sleep disorders, and poor school performance. In addition, it hinders social relationships and the development of emotional intelligence. To conclude, it is necessary to correctly use video games in particular, and technologies in general, adapting their content to children’s age, as well as the amount of time that they dedicate to use them.

## 1. Introduction

New technologies are blameworthy for the significant changes that society must face. We are in the digital age and technologies are progressing at an exponential rate, building our current situation [1]. But what do we consider a new technology? Roca defined new technology as everything that forms the set of information and communication technologies, video game consoles included [2].

Due to the COVID-19 pandemic, the average time children and adolescents spend playing video games or connected to the Internet has increased [3], as these devices become a platform to connect with other online users [4]. The pandemic restricted many of their recreational activities, so tablets, smartphones, computers, and game consoles became their only entertainment. Children and teenagers were using the Internet for many hours per day, without parents being able to control the use of these electronic devices [5].

### 1.1. Video Game Users

Children and teenagers are the main groups that play video games [6]. These groups attend primary education and secondary education.

Regarding primary education, some basic cognitive changes, which technologies could influence, can be highlighted. Students foster their chosen capacity consciously, try to achieve their objectives, their memory and attention capacities improve, and thinking becomes logical and flexible [7]. Moreover, at this age, they can identify their own emotions and others’ emotions, controlling and communicating them [8]. Empathy and self-esteem could increase or decrease depending on the social comparison with their peers; friendships play an important role, and technologies influence them [9].

On the other hand, students in secondary education experience different cognitive changes in comparison to primary school students. This stage represents a period of big changes [10]. In fact, during this phase, thinking turns abstract [11], a critical and reflexive attitude is developed which improves the capacity for problem-solving and the adequacy of social behavior, planning capacity is fostered, and adolescents’ thoughts focus on what they want but do not have [12]. In addition, on a social-affective level, teenagers have changeable emotional states; they need recognition from others such as social acceptance, they cannot express their feelings, and carry self-confidence problems [13].

### 1.2. Players’ Personality

Studies have revealed that there is a relationship between the playing style, the intensity of use, and the player’s personality [14]. Honesty, humility, openness to experience, conscientiousness, extraversion, agreeableness, and emotionality are considered personality dimensions [15].

In this sense, children and adolescents with low self-esteem, honesty, and/or humility choose to play violent games. They show psychopathic traits, and their gaming behaviors are aggressive [16]. For its part, adventure games are chosen by children and teenagers with a greater openness to experience [17]. They are also more successful in video games, which improves their self-efficacy, and stay engaged longer in these games [18]. 

Children and adolescents with high conscientiousness scores choose challenging and team-based games [14]. They achieve accomplishments that increase their self-reliance and keep them playing [18]. 

On the other side, users with extroverted personalities participate more in online environments and prefer survivor or daredevil games [19]. Thus, gamers who are more energetic and able to accept difficult challenges are more successful in video games [20]. In addition, high levels of friendliness by children and adolescents can lead to greater participation in video games, since in online games children play in teams, forming social bonds [21]. In fact, kinder people choose games that involve challenges to be solved in a cooperative way. 

Finally, children who are more anxious and dependent participate more in working on in-video games and professions because there is a lack of risk and failure [22]. Moreover, more emotional gamers prefer to replay different levels of the same game, to minimize depressing comments from others. On the other hand, more skilled players tend to be more emotionally calm when playing and choose mostly gambling games [18]. 

Video games are also used as an online tool to escape loneliness [23]. However, previous studies have also shown that a lack of self-control and autonomy may suppose a risk for future video game addiction problems [24]. In turn, children and adolescents who are unable to adequately manage their gaming time will have a higher risk of gambling addiction [25]. In addition, personality traits such as aggression or narcissism correlate positively with video game addiction [26]. 

### 1.3. Video Game Influence

In the technological environment, we can find video games. They have an essential role in children’s and adolescents’ life because they modify substantially their way of understanding, interacting, and communicating [27].

The video game market is the leading audiovisual and interactive leisure industry in our country, in fact, four out of five families have a gadget to play with at home [28]. These data demonstrate the high popularity of video games among the general population. 

Children and adolescents seem to have a great fascination for and attraction to games. The reason could be because videogames are more than entertainment [29]. Video games which have an important socializing role are a model of behavior for young people [30], and this model could be negative or positive depending on the game. In addition, video games are involved in identity construction [31].

### 1.4. Adverse Effects of Video Games

Before talking about the negative effects of excessive use of video games, we must differentiate between two concepts [32]: “internet addiction disorder (IAD)” and “internet gaming disorder (IGD)”. 

IAD is understood as “the excessive use of the Internet in an uncontrolled and time-consuming manner that leads to timelessness and disruption of people’s lives” [33] (p. 1). On the other hand, IGD is defined as “persistent and repeated use of the Internet to engage in gaming, usually with other users, that causes clinically significant impairment or distress” [34] (p. 1). If five or more criteria are met for 12 months, IGD is diagnosed. The criteria are preoccupation, tolerance, drawdown, persistence, escape, problems, disappointment, displacement, and conflict [35].

IGD is included as a disorder in the Diagnostic and Statistical Manual of Mental Disorders, fifth edition (DSM-5), but IAD is not included. On the contrary, both concepts cause negative impacts and provoke behaviors that could lead to psychological, social, and personal adversities [36].

Despite the fact that young people are considered digital natives [37], they are especially vulnerable to video games’ effects because they are growing up and do have not the full potential of their psychosocial resources [31]. For this reason, compulsive and excessive exposition to video games could modify the levels of healthy use and, consequently, provoke a disorder in the personal development of children and teenagers with effects on a social level or state of mind [38]. Thereby, supervision is a big defiance for families and responsible adults in our actual society [6].

Excessive participation in video games is associated with undesired behaviors such as stress, emotional changes, aggressive behaviors, hyperactivity, and impulsivity [39]. Furthermore, the frequency of use of video games encroaches on social interactions; it is associated with loneliness, retracted manners, and low self-esteem [40]. In the educational environment, students who spend a lot of time playing video games during schooldays perform worse academically [41]. In fact, a recent study affirms that academic achievement is influenced by game context [42].

We can summarize the negative effects of excessive use of video games as the following: reduced attention, emotional and social intelligence deterioration, social isolation, and sleep disorders [43].

In addition, gaming disorders are associated with deregulation of the dopamine reward system [44], poor long-term vision from prolonged screen time and dizziness [45], possible epileptic seizures due to the flashing and contrasting light in the animation [46], obesity problems from changing physical activity to electronic devices [47], and brain disorders related to concentration and thinking, so that children become apathetic [48]. What is more, they can cause headaches due to the electromagnetic radiation emitted by the screen [49] and physical, muscular and bone stress, as well as back or neck problems from sitting incorrectly in front of these games for long periods of time [50]. Furthermore, anxiety is one of the major symptoms of psychopathology associated with video games [51]. People may turn to video games to calm their anxiety, so they learn that video games are a way to avoid negative moods [52]. Thus, the immediate gratification offered by video games encourages children to use games when they experience negative situations. This action can become a habit and, therefore, video games are prioritized over other important aspects of life [53].

Even some recent studies show that children may experience cardiac arrhythmia during electronic games due to adrenergic stimulation related to the emotionally charged video game environment [54].

Regarding the importance of video games for young people, we focus on the adverse consequences in behavior because of violent and inadequate video games.

### 1.5. Positive Effects of Video Games

However, it is also necessary to consider the benefits of video, with the objective of understanding how video games impact the development of children and adolescents. The nature of video games has changed in recent years, becoming increasingly realistic and diverse [55]. For this reason, the potential benefits of video games must be contemplated. We focus on four main areas: cognitive, motivational, emotional, and social [56].

In respect of the cognitive domain, playing video games, especially action games, promotes a wide range of cognitive skills, such as faster and more accurate attention [57], higher spatial resolution in visual processing [58], better mental rotation abilities [59], greater spatial skills [60], more effective filtering of irrelevant information [57], more flexible problem solving [61], and greater development of creativity [62]. 

Regarding the motivational domain, video games provide players with immediate feedback that rewards effort and keeps players within what Vygotsky [63] called the “zone of proximal development”. This motivational point balances levels of challenge and frustration which causes experiences of success [64]. Thus, players face challenges with motivation and optimism to achieve their goals [65]. 

Respecting the emotional domain, previous studies show a causal relationship between playing video games and improving mood or increasing positive emotions. Children describe playing video games as a rewarding experience that provokes a sense of control, thus increasing their self-esteem [21]. 

Finally, on the subject of the social domain, the virtual social communities created in video games make children quickly learn social skills and prosocial behavior. In this sense, effective cooperation, support, and helping behaviors are developed [66]. Subsequently, these behaviors can be generalized to their friendship and family relationships outside the game environment [67].

### 1.6. Objectives

The overarching objective of this study is to investigate literature that directly relates the continuous and undifferentiated use of video games with the emergence of behavioral disorders in children and young people.

Specifying this aim, some hypotheses were raised:

**H_1_**. *Playing video games constantly is a risk factor; it could provoke technology addiction disorders*.

**H_2_**. *Use of technology and video games affect sleep*.

**H_3_**. *Aggressive video games foster aggressive behaviors in real life*.

**H_4_**. *Excessive use of video games makes social relationships more difficult*.

**H_5_**. *Excessive use of video games may provoke a lower academic performance*.

**H_6_**. *Excessive use of technologies and video games hinders emotional intelligence development*.

**H_7_**. *Playing video games provokes attention deficit and hyperactivity disorder (ADHD)*.

## 2. Materials and Methods

In this systematic review, the statement of the preferred reporting items for systematic review and meta-analyses 2020 (PRISMA statement) [68] was followed. The study, data, and materials included in this article were not preregistered. 

### 2.1. Literature Search

PubMed, Web of Science, and SCOPUS were the databases chosen. The searches were performed from September to November 2022, with the objective of compiling the largest list of studies we could. We looked for relevant articles about the effects of playing video games on behavioral disorders. Additionally, manual searches were carried out on the references of the studies selected and we identified potential studies [69].

Two Boolean operators were used: AND/OR. The descriptor words were “behavioral disorders”, “behavioral problems”, “video games”; they were used in different ways depending on the database. We did not apply any filters or limitations because we wanted to identify the greatest number of appropriate studies [70]. It is true that we had applied publication date filters at first; however, several relevant studies had been removed. 

In Table 1, the search strategy can be seen. 

### 2.2. Selection Process

Firstly, 113 articles were identified in an automatic search. They were imported to a Word file to identify duplicate articles. Out of 113, there were 5 duplicated. 

Secondly, we found relevant studies by abstract, keywords, and title. This phase was screened by two independent researchers to eliminate irrelevant articles, which resulted in 70 articles. They used the following exclusion and inclusion criteria:

Articles that included our keywords (behavioral problems, behavioral disorders, video games) were accepted. We refused those articles which did not talk about our topic.

Articles from scientific specialized journals were included. We refused studies in book chapters, doctoral theses and grey literature in general.

Articles with open access were included. We refused articles to which we did not have complete access.

Articles were required to be completed works; we refused in-process articles.

Participants were required to be children or young people; adults’ samples were refused.

A total of 38 articles were selected using these criteria.

**Figure 1:**
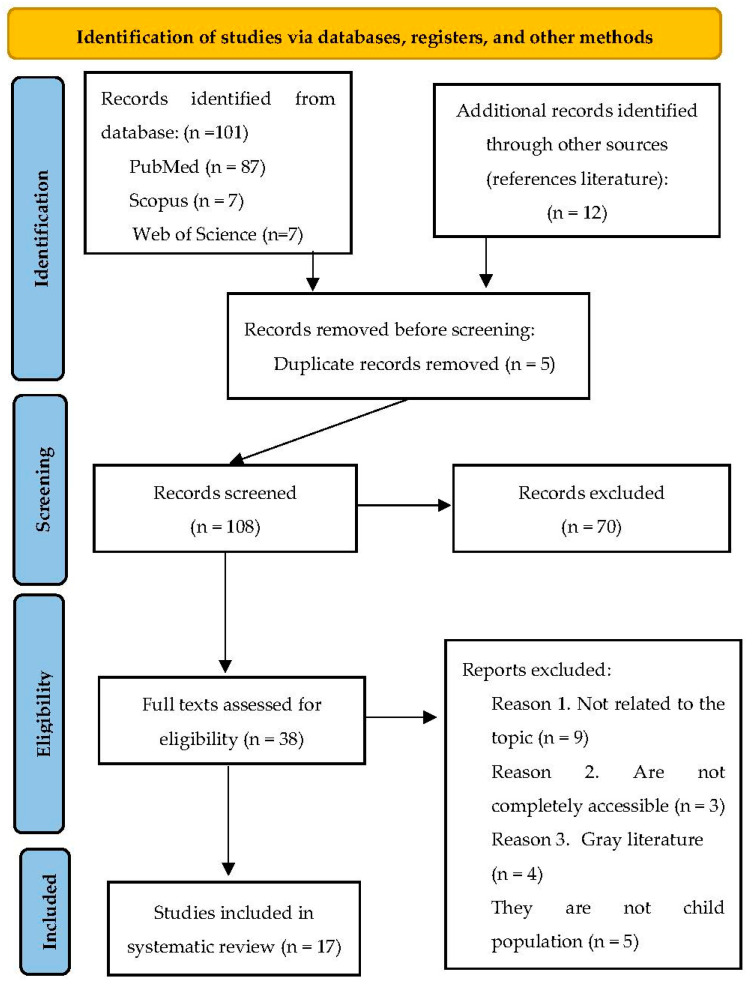
Flow diagram of the articles during the selection process.

Thirdly, the same two independent researchers assessed the articles chosen for eligibility by reading all the text. They applied the same criteria and found 17 relevant articles which were included in our systematic review. In Figure 1, the selection process can be seen.

### 2.3. Data Extraction

Seventeen studies were analyzed to extract data from them. In a table, two independent researchers compiled the names of authors, publication date, objective(s), the instrument used, sample, age of the sample, country, and outcomes. A third researcher validated the collected information. When some required information was not available, we asked the corresponding author to obtain it; however, sometimes we did not receive any reply. 

The instruments used included the variables measured and the age of the sample included the mean.

### 2.4. Study Quality Assessment

Descriptors were discussed between the authors who reached an agreement. Then, the selection process was carried out by two authors independently. Any disagreement between the two reviewers was solved by consensus. Dividing the number of agreements by the total number of disagreements plus agreements, we calculated the inter-rater reliability. Then we multiplied by 100 to get the percent. The inter-rater reliability was 94%.

## 3. Results

### 3.1. Study Characteristics

A total of seventeen articles were analyzed in detail. They were carried out across several countries around the world. Four were from the United States [71,72,73,74], two from China [75,76], two from Italy [77,78], and the rest of the countries were studied in one article each. The continent most studied was Asia, in eight out of seventeen articles, closely followed by America (four from North America [71,72,73,74], and one from South America [79]).

The age range was very large, from 6 months to 23 years old. However, the majority of the studies had a population aged from 9 to 15 years old. Regarding the instrument, they were varied. Questionnaires and interviews conducted by researchers of the study were the instruments used most (in 52,94% of the articles) [73,75,77,79,80,81,82,83,84], followed by the Child Behavior Checklist [79,80,85] and demographic data [76,77,84]. (See Supplementary Table S1 for characteristics of each article selected).

### 3.2. Findings

Before starting to analyze the influences on children of playing video games, we want to highlight the importance of considering the different types of games and devices that are included in the articles selected. In this sense, it is also necessary to specify the age-appropriateness, the use frequency, the timetable, or the time they spend gaming. For these reasons, we analyzed each article in detail.

This information is mentioned roughly in sociodemographic data in most of the articles; there are only a few that explain it in an explicit way. Moreover, Yousef et al. specify that the limitation of their work is there is no evaluation of the TV programs and video games. [80], Accordingly, Chindamo et al. mention that they did not register the time children spent playing [77]. 

Nevertheless, data found in some of the studies reveal important findings about the content of games and aggression or delinquent behaviors. For instance, Wei showed that playing violent video games is positively correlated with violence tolerance and aggressivity [83].

Regarding the age adequacy of each game used, Yilmaz et al. had a sample aged 9–10 years old and most of the games included in their investigation are for teenagers over 13 years old [83]. In this sense, Salih et al. used a sample aged 4–17 years old. Because of the wide range of children included, it was thought that they used different types of games. However, they used the same games for all participants. These three games are for people over 13 and 16 years old, respectively [84]. On the contrary, Kirsh and Mounts used adequate games for their sample age [77]. 

Similarly, the use frequency and the time children spend gaming are essential to determining a limit between adequate use and excessive use. Accordingly, Cheung et al. affirmed that more frequent use of digital screens provokes less sleep time. In numeric data, each additional hour of tablet use was associated with 15.6 min less total sleep [81]. Finally, the timetable for playing video games is so relevant. In fact, pre-sleep habits have a big impact on teenagers’ sleep [78]. Likewise, significant correlations between time spent playing video games just before bed and sleep disorders were demonstrated [86]. 

#### 3.2.1. Technology Addiction Disorders (TAD)

We find a positive correlation between the use of video games and technology addiction [76,84]. This has been exacerbated by COVID-19 [76]. Moreover, we observe close relationships between TAD and ADHD [82,85,87], and anxiety [76]. In addition, the number of hours that young people spend playing video games provokes attention problems [80,84,86]. (See Table 2 for more specific information).

#### 3.2.2. Sleep Disorders

We observed in Table 3 a negative relationship between playing time and sleep duration [77,78,81,86]. In addition, we found that gaming frequency significantly increased sleep onset latency [77,81]. In addition, children and adolescents who spend a lot of time playing video games have sleep disorders in general [84]. However, Cheung et al. did not find a significant correlation between the use of a digital screen and the frequency of nocturnal awakenings. Likewise, we find a close relationship between playing video games and going to bed late [78,87].

#### 3.2.3. Aggressive Behavior 

We find a close relationship between the number of hours playing video games per day and the occurrence of aggressive and delinquent behaviors [73,75,80,82]. In turn, we observe a significant positive correlation between exposure to violent video games and attitudes toward violence [75,84]. Furthermore, reduced happy-face advantage can be found because of violent games [72]. Finally, self-control correlates negatively and significantly with hours spent playing video games [73,82]. Table 4 details this information.

#### 3.2.4. Academic Performance

We find a negative correlation between academic performance and hours playing video games [73]. Likewise, problematic behavior at school is positively and significantly correlated with hours playing video games [73]. On the other hand, hardcore gamers have to be helped with homework by other classmates [82]. These gamers use video games during school days and prefer them instead of studying [84]. On the contrary, Yilmaz et al. [82] affirmed that some teachers claim that playing video games helps to learn English. In addition, Rodríguez and Sandoval [79] disagreed; they did not find significant differences between the use of video games and academic performance. 

#### 3.2.5. Emotional Intelligence Development

We observe a close relationship between anxiety symptoms in adolescents and abusive use of video games; however, there are no significant differences in children [76]. On the other hand, depressive symptoms have no significant differences in any age group [76]. Furthermore, we find that hardcore gamers have affective problems [82]. Regarding gender, boys enjoy and feel more excitement when playing video games than girls [72]. Finally, we observe a close relationship between playing violent video games and a reduced expression of happiness [72].

#### 3.2.6. Attention and Hyperactivity Disorders

We find a negative relationship between playing time and sustained attention [86]. In addition, it is claimed that hardcore gamers have attention problems in class [82]. This relationship between video games and sustained attention was measured by sleep duration [86]. 

It is observed that video games predispose players to hyperactivity [84]. Nevertheless, it is true that children with ADHD had higher levels of Internet addiction and spent more hours using video games [87]. To sum up, we can say that Internet addiction is positively and significantly related to inattention, hyperactivity–impulsivity, and total attention deficit hyperactivity disorder [85].

#### 3.2.7. Social Relations

Despite the fact that social relationships were not in our minds when we started to investigate video games and technologies, several results emerge from the articles selected (Table 5).

We find a positive correlation between time spent playing video games and social problems [80]. In addition, hardcore gamers had trouble relating to their classmates, especially girls, because of the topics of conversation. However, communication among hardcore gamers is solid [82]. Finally, we observe a close relationship between playing violent video games and a lack of empathy (negative relation) [75] and competitive behaviors (positive relation) [71]. 

## 4. Discussion

This systematic review has given an analysis of the effects of video games in several areas of children’s and teenagers’ lives. Through this epigraph, we analyze the hypothesis we raised before.

In this way, we can observe that H_1_ “playing video games constantly is a risk factor, it could provoke technology addiction disorders” is confirmed by Teng et al. [76] and Salih et al. [84], whose results show that video games predisposed players to addictive effects. In turn, other studies supported it, they found a significant difference between the time spent playing video games and the addiction. [88]. Not only in children and adolescents but also in adults Internet addiction is found, with differences depending on age; younger ones have more risk of being addicted [89]. Moreover, we found that COVID-19 has increased the use of video games and the addiction [4,76]. This statement is supported by the authors of [90,91,92]. The reason for this finding is the lockdown. People had more free time and they played video games more frequently [93]. Considering gender, it was studied that boys have a higher tendency to engage in and become addicted to video games [94].

The Interaction of Person–Affect–Cognition–Execution (I-PACE) explains the predisposing variables for problematic internet behavior (addiction, gaming, etc.) [53]. It includes genetic, biological, personality, cognitive and motivation factors, and psychopathology variables (such as depressive and anxiety symptoms) as predisposing variables. Moreover, the WHO recently included Internet Gaming Disorder as a mental health disorder [95]. 

COVID-19 has impacted aspects of human life in general, but one of the key research priorities was mental health [96]. Chronic stress during the lockdown has generated negative emotional distress such as depression or anxiety [97,98] and children are more susceptible to engaging in video game playing to deal with these adverse feelings [91,99]. This engagement could predispose children to experience internet gaming disorder as a stress response [100] since depressive and anxiety symptoms are predisposing variables affecting gaming [101].

Regarding H_2_, we found a major number of articles that studied the relationship between the use of technology or video games and sleep disorders [76,77,81,83,84,86,87]. Sleep time is reduced [77,78,81,86], and sleep onset latency is increased [77,81] when people use daily technologies. Specifically, for video games, Wolfe et al. [86] said that there is a significant and negative correlation between gaming time and sleep duration. Other authors agreed with our results, finding that playing games for a long time during the day decreases sleep duration and quality [102,103,104]. On the other hand, Cheung et al. [81] found that there was not a significant correlation between digital screen use and the frequency of night awakenings. Meanwhile, Fobian et al. [105] disagree. For these reasons, parental limit-setting is so important [106].

H_3_ is related to aggressive behaviors. The American Psychiatric Association affirms that scientific research has demonstrated a close relation between violent video games and increases in aggressive cognitions, behaviors, and affect. [107]. Our findings support that video games foster undesired behaviors, and violent video games foster aggressive behaviors [72,73,80,82] and violence [75,84]. Hasan et al [108] confirm that violent video games are associated with higher aggression levels [109], and Greitemeyer and Mügge [110] say that prosocial games are associated with lower aggression levels. 

Society frames aggression in negative terms [111]. It is defined as a behavior that is intended to improve one’s own social dominance at the expense of another [112]. For its part, violence could not be taken as a synonym for aggression. Violence is not always framed in negative terms since violent acts could be motivated by self-defense or the defense of someone [112]. While aggression could be innate [113,114], violence is intentional. The World Health Organization defined it as “the intentional use of physical force or power against oneself or another person, or against a group or community, that either results in or has a high likelihood of resulting in injury, death, psychological harm, maldevelopment or deprivation” [115] (p. 3). Moreover, we also found that spending more than 2 hours per day watching TV or playing video games, had higher scores on delinquent behavior [80]. This delinquent behavior is related to criminality. In this case, it is juvenile delinquency because the sample is under 18 years old, and it is defined as actions against the criminal laws of each country [116]. 

Furthermore, significant differences were found between genders. Boys enjoyed playing video games more than girls [72]. Females felt video games were frustrating. Moreover, boys behave more aggressively than girls after playing violent video games [117] because males display significantly higher aggressive cognition [118].

These behaviors provoke difficulties in social relationships. Our hypothesis (H_4_) is corroborated by our findings [71,80,82]. It is also verified by Robertson et al. [119], who affirm the relationship between screen time and antisocial behavior in early adulthood. On the other hand, Kuss y Griffiths [120] affirms the effects in social behavior impact just in intensive video gamers.

Low social relationships and aggressive behaviors have a big impact on the educational environment. Teachers and classmates cannot relate well with hardcore gamers [80] and attention is lost [84]. The aforementioned issues affect academic performance. Chen et al [121] and Adelantado-Renau et al. [122] found a significant and negative relationship between the hours spent playing video games and the general score. In line with them are our results [73,82,84]. Thus, we can say that H_5_ is corroborated too; excessive use of video games provokes a lower academic performance in the different educative systems of the countries studied.

Emotional intelligence is also important in school performance because it is a predictor of scores of more importance than standardized tests [123]. Our H_6_ says that excessive use of technologies and video games hinders emotional intelligence development, and our results corroborate it [72]. These results are in line with previous studies that show a negative correlation between time spent playing video games and self-esteem and satisfaction with life [124]. Likewise, another study shows that exposure to violent video games is negatively related to the accuracy of recognition of negative emotions in others’ faces, [125]. Our results also show a decrease in empathy when people play violent video games [75], which is ratified by Díaz et al. [126].

Finally, H_7_ says “playing video games provokes attention deficit and hyperactivity disorders (ADHD). At first, it was rejected because we found that ADHD was the cause of video game addiction [87]. However, we also found a negative relationship between playing time and sustained attention or attention problems [82,85,86]. Moreover, video games predispose players to hyperactivity [84]. In this sense, numerous authors affirm our results; there is a significant association between playing video games, Internet addiction, and ADHD [88,127]. In fact, pathological Internet use is correlated with social anxiety disorder, anxiety, obsessive–compulsive symptoms, and depression [128]. All of these are possible characteristics of ADHD. Moreover, it was affirmed that the child group most likely to be addicted is ADHD students, even more than students with other psychiatric symptoms [129]. 

Some main ADHD symptoms predict these types of disorders, such as “being easily bored” or “having an aversion for delayed rewards” [130]. These people have an impulsive need for rapid satisfaction. Core characteristics of ADHD could explain the relationship between this disorder and the Internet and gaming addiction. [131] In fact, the severity of ADHD symptoms is associated with the severity of IGD [132]. Using the Internet provides several activities at the same time and instant rewards which could decrease the symptoms mentioned and make ADHD children become addicted to the internet [133]. Apart from that, abnormal brain activities were found in this population. They produce impaired inhibition, which reduces self-control ability and fosters the incapacity to restrain themselves. Likewise, these people are more vulnerable to internet addiction and video game use [133]. 

**Figure 2:**
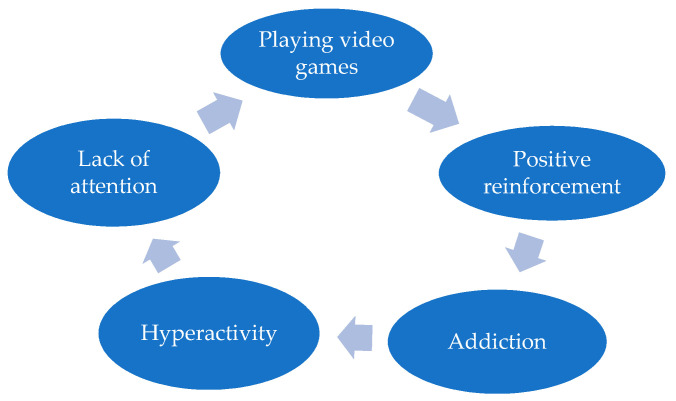
The cyclical process of ADHD and video games use.

Relating the two concepts, Weiss et al. [134] affirm that there is a bidirectional relationship between ADHD and Internet addiction or addiction to playing video games. They say that ADHD makes games more attractive, and games aggravate the symptoms of ADHD (See Figure 2). This could be explained by the necessity of ADHD children to escape from their social and emotional problems and stay in a place without obligations [135]. 

## 5. Conclusions

Technologies have a strong impact on people’s lives, in general; however, the groups of people who are more affected by them are children and teenagers. 

The main objective of this study was to find the effects of using video games in a continuous and undifferentiated way on behavioral disorders in children and young people. As we verified, using video games cause a lot of problems, not only in addition to the Internet or technologies but also in children and young people’s behaviors, social relationships, academic performance, sleep disorders, and so on.

PRISMA methodology is a good method in social science research because it makes studies transparent and open to suggestions and comments. However, our review has one limitation. We refused grey literature, such as other systematic reviews. We selected exclusively articles from specialized journals because we wanted scientific rigor in our article.

Finally, this article would be ideal for families and teachers of primary and secondary education, so they can understand what happens with their children if they do not control the use and type of video games. In this sense, they can use parental controls to avoid excessive use of these devices and inappropriate video games. Parents and guardians can control what children and adolescents are doing without being present. The Entertainment Software Ratings Board website has different manuals explaining how to restrict video game use, depending on the types of devices and software [136]. Blocking games that include inappropriate content or blocking them by age rating and limiting the time children play would be very recommended. On the other hand, TcosMonitor is an app that teachers can use to control the devices inside their classrooms from their own computers. Not only for control but also for help, teachers can access children’s desktops and manipulate them [137]. 

## Figures and Tables

**Table 1:** Search strategy for each source.

Source	Specificities of Each Database	Search Strategy	Number of Articles Found
SCOPUS	Article title, Abstract, Keywords	(“behavioral disorders” OR “behavioral problems”) AND “videogames”	7
Web Of Science	Topic (article title, abstract, keywords, keywords plus)	(“behavioral disorders” OR “behavioral problems”) AND “videogames”	7
PubMed		(“behavioral disorders OR “behavioral problems”) AND “videogames”	87
References literature		-	12

**Table 2:** Results related to technology addiction disorders.

Authors	Results
Yoo et al., 2004 [85]	- The ADHD group had significantly higher addiction than the non-ADHD group (*p* < 0.001). - Men had higher levels of Internet addiction than women (*p* < 0.01) - Internet addiction was positively and significantly related to inattention (*p* < 0.001), hyperactivity–impulsivity (*p* < 0.001), and total attention deficit hyperactivity disorder (*p* < 0.01).
Wolfe et al., 2014 [86]	- There is a significant and negative correlation between gaming time and sustained attention (*p* < 0.01).
Yousef et al., 2014 [80]	- Children who spent more than 2 h per day watching TV or playing video games had higher scores on attention problems (*p* = 0.002)
Weinstein et al., 2015 [87]	- Children with ADHD had higher levels of Internet addiction than children without ADHD diagnosis, with significant differences (*p* < 0.01). - ADHD group spent more hours using the Internet or video games (*p* < 0.05).
Yilmaz et al., 2018 [82]	- Teachers affirmed that hardcore gamers had attention problems and were more aggressive.
Salih et al., 2020 [84]	- Video games predisposed players to lack of attention (23%) and addictive effect (5.9%).
Teng et al., 2021 [76]	- Video game use (*p* < 0.001), increased exponentially after the COVID-19 pandemic in both, children and adolescents. - Anxiety symptoms are correlated with Internet gaming disorders (*p* < 0.001).

**Table 3:** Results related to sleep disorders.

Authors	Results
Wolfe et al., 2014 [86]	- There is a significant and negative correlation between gaming time and sleep duration (*p* < 0.001). - Older teenagers spent less time video gaming (*p* < 0.05) and spent more time sleeping (*p* < 0.05).
Weinstein et al., 2015 [87]	- ADHD group went to sleep later, most of them after midnight. - Results showed a relationship between ADHD, sleep disorder, and Internet/video games addiction.
Cheung et al., 2017 [81]	- A significant and positive association between digital screen use and sleep time during the day (*p* < 0.05) can be observed. Sleep onset (*p* < 0.001) was positively related to digital screen use. On the contrary, the relation between the digital screen and sleep time at night is negative (*p* < 0.001). - There was not any significant correlation between digital screen use and the frequency of night awakenings - An increase in digital screen use was associated with a decrease in the overall amount of sleep.
Chindamo et al., 2019 [77]	- Frequency of video game playing had a significative impact on sleep onset latency (*p* < 0.005), increasing the time to get to sleep, but the differences in total sleep time were not significative although it was reduced. - Daily tablet or mobile use reduced the total sleep time (*p* < 0.05) and increased the sleep onset latency (*p* < 0.05).
Salih et al., 2020 [84]	- Video games predisposed players to sleep disorders (45%).
Nosetti et al., 2021 [78]	- There was a negative correlation between the use of mobile phones (*p*= 0.03) or the Internet (*p* < 0.01) and pre-sleep habits and sleep time. - Use of mobile phones (*p* = 0.003) or the Internet (*p* = 0.008), playing video games (*p* = 0.009), and listening to music (*p* = 0.001) as pre-sleep habits had a positive correlation to late bedtime.

**Table 4:** Results related to aggressive behavior.

Authors	Results
Kirsh and Mounts, 2007 [72]	- Students who played violent video games presented a significantly reduced happy-face advantage (*p* < 0.05).
Wei, 2007 [75]	- Adolescents who spent more time playing violent video games were more pro-violence and less concerned about others. There was a significant and positive correlation between exposure to violent video games and attitudes to violence (*p* < 0.01). - The relationship between exposure to violent video games and aggression was positive and significant (*p* < 0.01).
Sharif et al., 2010 [73]	- School problem behavior is positively and significatively correlated to hours viewing TV, hours playing video games, and having a TV in the bedroom (*p* < 0.0001). - Self-control is negatively and significantly correlated to hours viewing TV (*p* < 0.0001), and hours playing video games (*p* < 0.0001).
Yousef et al., 2014 [80]	- Children who spent more than 2 hours per day watching TV or playing video games, had higher scores on aggression (*p* = 0.018) and delinquent (*p* = 0.023) behavior.
Yilmaz et al., 2018 [82]	- Following teachers’ and classmates’ answers, it is identified that HG had affective, verbal (mockery, ridicule), self-control, and behavioral (intimidation and physical aggression) problems. - Teachers affirmed that HG had attention problems.
Salih et al., 2020 [84]	- Violence and aggressive behavior were associated with video games in 25% of the sample.

**Table 5:** Results related to social relations.

Authors	Results
Sheese and Graziano, 2005 [71]	- Playing violent video games fosters competitive behaviors and has negative effects on social behaviors too. - Playing violent video games can affect later decisions to cooperate or compete with others in high-risk situations.
Wei, 2007 [75]	- There was a significant but negative correlation between playing violent video games and empathy (*p* < 0.001).
Yousef et al., 2014 [80]	- Children who spent more than 2 h per day watching TV or playing video games, had higher scores on being withdrawn (*p* = 0.001), and social problems (*p* = 0.3). - Following teachers’ and classmates’ answers, it is identified that HG had verbal (mockery, ridicule), self-control, and behavioral (intimidation and physical aggression) problems.
Yilmaz et al., 2018 [82]	- Girls found it difficult to communicate with hardcore gamers (HG) because of the topics of conversation. Teachers had poor communication with HG. Communication between HG is solid.

## Data Availability

Not applicable.

## Supplementary Materials

The following supporting information can be downloaded at: https://www.mdpi.com/article/10.3390/children10010086/s1, Table S1: Characteristics of Analyzed Studies [138].

## References

[1] Acerbi A. (2020). Cultural Evolution in the Digital Area.

[2] Roca R. (2018). Knowmads. Los Trabajos Del Futuro.

[3] Elsayed W. (2021). Covid-19 pandemic and its impact on increasing the risks of children’s addiction to electronic games from a social work perspective. Heliyon.

[4] Li Y.Y., Sun Y., Meng S.Q., Bao Y.P., Cheng J.L., Chang X.W., Ran M.S., Sun Y.K., Kosten T., Strang J. (2021). Internet Addiction Increases in the General Population During COVID-19: Evidence From China. Am. J. Addict.

[5] Parker K., Uddin R., Ridgers N.D., Brown H., Veitch J., Salmon J., Arundell L. (2021). The use of digital platforms for adults’ and adolescents’ physical activity during the COVID-19 pandemic (our life at home): Survey study. J. Med. Internet Res..

[6] Sans D.J. (2019). Adolescence and consumption of video games: A narrative review of the state of the art. Anu. Investig..

[7] Peña M.J., Aguaded E.M. (2019). Evaluación de la Inteligencia Emocional en el alumnado de Educación Primaria y Educación Secundaria. Rev. EUG.

[8] Mayer J.D., Caruso D.R., Salovey P. (2016). The ability model of emotional intelligence: Principles and updates. Emot. Rev..

[9] Carswell J.M., Stafford D.E., Neinstein L.S., Gordon C.M., Katzman D.K., Rosen D.S., Woods E.R. (2008). Normal physical growth and development. Adolescent Health Care. A Practical Guide.

[10] Moshman D. (2005). Adolescent Psychological Development. Rationality, Morality, and Identity.

[11] Fernández-Barba F.J., Heredia-Escorza Y. (2018). El desarrollo de la competencia de liderazgo en adolescentes en la ciudad de Tijuana. Perspect. Empresarial.

[12] Hidalgo M.I., Redondo A.M., Castellano G. (2012). Medicina de la Adolescencia. Atención Integral.

[13] Güemes M., Hidalgo M.I., Ceñal M.J. (2017). Desarrollo durante la adolescencia. Aspectos físicos, psicológicos y sociales. Pediatr. Integral..

[14] Bean A., Groth-Marnat G. (2016). Video games and personality: A five-factor model to understand game playing style. Psychol. Pop. Media Cult..

[15] Zeigler-Hill V., Monica S. (2015). The HEXACO model of personality and video game preferentes. Entertain. Comput..

[16] Worth N.C., Book A.S. (2015). Dimensions of video game behavior and their relationships with personality. Comput. Hum. Behav..

[17] Jhonson D., Gardnerb J., Sweetser P. (2016). Motivations for videogame play: Predictors of time spent playing. Comput. Hum. Behav..

[18] Teng C.I. (2008). Personality differences between online game players and nonplayers in a student sample. Cyberpsychol Behav..

[19] Braun B., Stopfer J.M., Müller K.W., Beutel M.E., Egloff B. (2016). Personality and video gaming: Comparing regular gamers, non-gamers, and gaming addicts and differentiating between game genres. Comput. Hum. Behav..

[20] Fortes G., Valtchanov D., Reetz A., Wehbe R.R., Orji R., Nacke L.E. (2018). Towards a trait model of video game preferences. Int. J. Hum. Comput. Interact.

[21] Ryan R.M., Rigby C.S., Prybylski A. (2006). The motivational pull of video games: A self-determination theory approach. Motiv. Emot..

[22] Worth N.C., Book A.S. (2014). Personality and behavior in a massively multiplayer online role-playing game. Comput. Hum. Behav..

[23] Amichai-Hamburger Y., Ben-Artzi B. (2008). Loneliness and Internet use. Comput. Hum. Behav..

[24] Mihara S., Higuchi S. (2017). Cross-sectional and longitudinal epidemiological studies of Internet gaming disorder: A systematic review of the literature. Psychiatry Clin. Neurosci..

[25] Chen I.H., Lee Z.H., Dong X.Y., Gamble J.H., Feng H.W. (2020). The Influence of Parenting Style and Time Management Tendency on Internet Gaming Disorder among Adolescents. Int. J. Environ. Res. Public Health.

[26] Kim E.J., Namkoong K., Ku T., Kim S.J. (2007). The relationship between online game addiction and aggression, self-control and narcisistic personality traits. Eur. Psychiatry.

[27] Peeters M., Koning I., van de Ejinden R. (2018). Predicting Internet Gaming Disorder symptoms in young adolescents: A one-year follow-up study. Comput. Hum. Behav..

[28] Asociación Española de Videojuegos. http://www.aevi.org.es/consumo-global-videojuegos-espana-supero-los-1-000-millones-euros-2015/.

[29] González J.C., Gramigna A. (2009). Comunicación joven: A propósito de la fascinación y la prestación educativa de los nuevos medios. Miscelánea.

[30] Isidro A.I., Berciano M., Castejón J.L. (2016). Consumo de videojuegos y su influencia en adolescentes. Psicología y Educación: Presente y Futuro.

[31] Sánchez J.I., Benítez E.I. (2022). Revisión sobre la “salud mental y nuevas tecnologías”. Análisis de las redes sociales y los videojuegos en las primeras etapas de desarrollo como factores modulares de una salud mental positiva. Int. J. Dev. Educ. Psychol..

[32] El Fiky R., Mansour M., Fekry M., ElHabiby M., Elkholy H., Morsy M. (2022). Occurrence of problematic Internet use and its correlates among Egyptian adolescent students in international schools in Cairo. Middle East Curr. Psychiatry.

[33] Sayed M., Naiim C.M., Aboelsaad M., Ibrahim M.K. (2022). Internet addiction and relationships with depression, anxiety, stress and academic performance among Egypt pharmacy students: A cross sectional designed study. BMC Public Health.

[34] Feng W., Ramo D.E., Chan S.R., Bourgeois J.A. (2017). Internet gaming disorder: Trends in prevalence 1998-2016. Addict Behav..

[35] Lemmens J., Hendriks S.J.F. (2016). Addictive Online Games: Examining the Relationship Between Game Genres and Internet Gaming Disorder. Cyberpsychol Behav. Soc. Netw..

[36] Kim K.M., Kim H., Choi J.W., Kim S.Y., Kim J.G. (2020). What types of Internet Services Make Adolescents Addicted? Correlates of Problematic Internet Use. Neuropsychiatr Dis. Treat.

[37] Trung T., Manh-Toan H., Thanh-Hang P., Minh-Hoang N., Khanh-Linh P.N., Thu-Trang V., Thanh-Huyen T.N., Thanh-Dung N., Thi-Linh N., Khuc Q. (2020). How Digital Natives Learn and Thrive in the Digital Age: Evidence from an Emerging Economy. Sustainability.

[38] Masfety V.K., Keyes K., Hamilton A., Hanson G., Bitfoi A., Golitz D., Koç C., Kujipers R., Lesinskiene S., Mihova Z. (2016). Is time spent playing video games associated with mental health, cognitive and social skills in young children?. Soc. Psychiatry Psychiatr Epidemiol..

[39] Moncada J., Chacón Y. (2012). El efecto de los videojuegos en variables sociales, psicológicas y fisiológicas en niños y adolescentes. Retos. Nuevas Tend. En Educ. Física Deporte Y Recreación.

[40] Rodríguez M., García F.M. (2020). El uso de videojuegos en adolescentes. Un problema de Salud Pubública. Enfermería Glob..

[41] Carrillo P.J., García M. (2022). Consumo habitual de videojuegos y rendimiento académico en escolares de primaria. Educ Knowl. Soc..

[42] Gómez F., Devís J., Molina P. (2020). El tiempo de uso de los videojuegos en el rendimiento académico de los adolescentes. Rev. Científica Iberoam. De Comun. Y Educ..

[43] Small G.W., Lee J., Kaufman A., Jalil J., Siddarth P., Gaddipati H., Moody T.D., Bookheimer S. (2020). Brain health consequences of digital technology use. Dialogues Clin. Neurosci.

[44] Kuss D.J., Pontes H.M., Griffiths M.D. (2018). Neurobiological correlates in internet gaming disorders: A systematic literature review. Front. Psychiatry.

[45] Laato S., Laine T.H., Islam A.K.M. (2020). Location-based games and the COVID-19 pandemic: An analysis of responses from game developers and players. Multimodal. Technol. Interact.

[46] Bahrilli T., Hamiyet Y., Çakir Y.N. (2020). Determining the health problems of electronic athletes. Asian J. Empir. Res..

[47] Dunton G.F., Do B., Wang S.D. (2020). Early effects of the COVID-19 pandemic on physical activity and sedentary behavior in children living in the US. BMC Publ Health.

[48] Mundy L.K., Canterford L., Hog M., Olds T., Moreno-Betancur M., Sawyer S., Patton G.C. (2020). Electronic media use and academic performance in late childhood: A longitudinal study. PLoS ONE.

[49] Wang X., Hedge S., Son C., Keller B., Smith A., Sasangohar F. (2020). Investigating mental health of US college students during the COVID-19 pandemic: Cross-sectional survey study. J. Med. Internet Res..

[50] Barbero E.M., Carpenter D.M., Maier J., Tseng D.S. (2018). Healthcare encounters for Pokemon go: Risks and benefits of playing. Game Health J..

[51] Bargeron A.H., Homes J.M. (2017). Psychosocial correlates of internet gaming disorder: Psychopathology, life satisfaction, and impulsivity. Comput. Hum. Behav..

[52] Brand M., Young K.S., Laier C., Wölfing K., Potenza M.N. (2016). Integrating psychological and neurobiological considerations regarding the development and maintenance of specific Internet-use disorders: An Interaction of Person-Affect-Cognition-Execution (I-Pace) model. Neurosci Biobehav. Rev..

[53] Brand M., Wegmann E., Stark R., Müller A., Wölfing K., Robbins T.W. (2019). The interaction of person-affect-cognition-execution (I-Pace) model for addictive behaviors: Update, generalization to addictive behaviors beyond internet-use disorders, and specification of the process character of addictive behaviors. Neurosci Biobehav. Rev..

[54] Nah D., Lee H.R., Janson C., Richardson-Olivier C., Shah M.J. (2020). Video game ventricular tachycardia: The “Fornite” phenomenon. HeartRhythm Case Rep..

[55] Ferguson C.J., Olson C.K. (2013). Friends, fun, frustation and fantasy: Child motivations for video game play. Motiv. Emot..

[56] Granic I., Lobel A., Engels R. (2014). The Benefits of Playing Video Games. Am. Psychol..

[57] Bavelier D., Achtman R.L., Mani M., Föcker J. (2012). Neural bases of selective attention in action video game players. Vis. Res..

[58] Bavelier D., Davidson R.J. (2013). Brain training: Games to do you good. Nature.

[59] Green C.S., Bavelier D. (2012). Learning, attentional control, and action video games. Curr. Biol..

[60] Uttal D.H., Meadow N.G., Tipton E., Hand L.L., Alden A.R., Warren C., Newcombe N.S. (2013). The malleability of spatial skills: A meta-analysis of training studies. Psychol. Bull..

[61] Adachi P.J., Willoughby T. (2013). More than just fun and games: The longitudinal relationships between strategic video games, self-reported problem solving skills, and academic grades. J. Youth Adolesc..

[62] Jackson L.A., Witt E.A., Games A.I., Fitzgerald H.E., von Eye A., Zhao Y. (2012). Information technology use and creativity: Findings from the Children and Technology Project. Comput. Hum. Behav..

[63] Vygotsky L. (1978). Mind in Society: The Development of Higher Psychological Functions.

[64] Salminen M., Ravaja N. (2008). Increased oscillatory theta activation evoked by violent digital game events. Neurosci. Lett..

[65] McGonigal J. (2011). Reality is Broken: Why Games Make up Better and How They can Change the World.

[66] Ewoldsen D.R., Eno C.A., Okdie B.M., Velez J.A., Guadagno R.E., DeCoster J. (2012). Effect of playing violent video games cooperatively or competitively on subsequent cooperative behavior. Cyberpsychol. Behav. Soc. Netw..

[67] Gentile D.A., Anderson C.A., Yukawa S., Ihori N., Saleem M., Ming L.K., Sakamoto A. (2009). The effects of prosocial video games on prosocial behaviors: International evidence from correlational, longitudinal, and experimental studies. Personal. Soc. Psychol. Bull..

[68] Page M.J., McKenzie J.E., Bossuyt P.M., Boutron I., Hoffmann T.C., Mulrow C.D., Shamseer L., Tetzlaff J.M., Akl E.A., Brennan S.E. (2021). The PRISMA 2020 Statement: An Updated Guideline for Reporting Systematic Reviews. BMJ.

[69] Hutchings J., Martin-Forbes P., Daley D., Williams M.E. (2013). A randomized controlled trial of the impact of a teacher classroom management program on the classroom behavior of children with and without behavior problems. J. Sch. Psychol..

[70] Wong S.S.-L., Wilczynski N.L., Haynes R.B. (2006). Developing Optimal Search Strategies for Detecting Clinically Sound Treatment Studies in EMBASE. J. Med. Libr. Assoc..

[71] Sheese B.E., Graziano W.G. (2005). Deciding to Defect. The Effects of Video-Game Violence on Cooperative Behavior. Psychol. Sci..

[72] Kirsh S.J., Mounts J.R.W. (2007). Violent video game play impacts facial emotion recognition. Aggress Behav..

[73] Sharif I., Wills T.A., Sargent J.D. (2010). Effect of Visual Media Use on School Performance: A Prospective Study. J. Adolesc. Health.

[74] Horowitz-Kraus T., Hutton J.S. (2017). Brain connectivity in children is increased by the time they spend reading and decreased by the length of exposure to screen-based media. Acta Pediatri.

[75] Wei R. (2007). Effects of playing violent videogames on Chinese adolescents’ pro-violence attitudes, attitudes toward others, and aggressive behavior. Cyberpsychol. Behav..

[76] Teng Z., Pontes H.M., Nie Q., Griffiths M.D., Guo C. (2021). Depression and anxiety symptoms associated with internet gaming disorder before and during the COVID-19 pandemic: A longitudinal study. J. Behav. Addict..

[77] Chindamo S., Buja A., DeBattisti E., Terraneo A., Marini E., Gómez L.J., Marconi L., Baldo V., Chiamenti G., Doria M. (2019). Sleep and new media usage in toddlers. Eur. J. Pediatr..

[78] Nosetti L., Lonati I., Marelli S., Salsone M., Sforza M., Castelnuovo A., Mombelli S., Masso G., Ferini-Strambi L., Agosti M. (2021). Impact of pre-sleep habits on adolescent sleep: An Italian population-based study. Sleep Med..

[79] Rodríguez H.G., Sandoval C.M. (2011). Consumo de videojuegos y juegos para computador: Influencias sobe la atención, memoria, rendimiento académico y problemas de conducta. Suma Psicológica.

[80] Yousef S., Eapen V., Zoubeidi T., Mabrouk A. (2014). Behavioral correlation with television watching and videogame playing among children in the United Arab Emirates. Int. J. Psychiatry Clin. Pract..

[81] Cheung C.H.M., Bedford R., De Urabain I.R.S., Karmiloff-Smith A., Smith T.J. (2017). Daily touchscreen use in infants and toddlers in associated with reduced sleep and delayed sleep onset. Sci. Rep..

[82] Yilmaz E., Yel S., Griffiths M.D. (2018). The Impact of Heavy (Excessive) Video Gaming Students on Peers and Teachers in the School Environment: A Qualitative Study. Turk. J. Addict..

[83] Cabré-Riera A., Torrent M., Donaire-Gonzalez D., Vrijheid M., Cardis E., Guxens M. (2019). Telecommunication devides use, screen time and sleep in adolescents. Environ. Res..

[84] Salih E.M.M., Alghamdi A.H., Alzahrani A.Y.B., Alghamdi H.A.D., Alghamdi F.A.S., Alzubaidy A.S.M. (2020). Prevalence and Negative impact of Videogames among children and adolescents in Albaha citi, KSA. Med. Sci..

[85] Yoo H.J., Cho S.C., Ha J., Yune S.K., Kim S.J., Hwang J., Chung A., Sung H.S., Lyoo K. (2004). Attention deficit hyperactivity symptoms and Internet Addiction. Psychiatry Clin. Neurosci..

[86] Wolfe J., Kar K., Perry A., Reynolds C., Gradisar M., Short M.A. (2014). Single night video-game use leads to sleep loss and attention deficits in older adolescents. J. Adolesc..

[87] Weinstein A., Weizman A. (2012). Emerging Association Between Addictive Gaming and Attention-Deficit/Hyperactivity Disorder. Curr. Psychiatry Rep..

[88] Chan P.A., Rabinowitz T. (2006). A cross-sectional analysis of video games and attention deficit hyperactivity disorder symptoms in adolescents. Ann. Gen. Psychiatry.

[89] Andreassen C.S., Billieus J., Griffiths M.D., Kuss D.J., Demetrovics Z., Mazzoni E., Pallesen S. (2017). La relación entre el uso adictivo de las redes sociales y los video juegos y síntomas de trastornos psiquiátricos: Un estudio transversal a gran escala. RET.

[90] Abel T., McQueen D. (2020). The COVID-19 pandemic calls for spatial distancing and social closeness: Not for social distancing. Int. J. Public Health.

[91] King D.K., Delfabbro P.H., Billieux J., Potenza M.N. (2020). Problematic online gaming and the COVID-19 pandemic. J. Behav. Addict..

[92] Kiraly O., Potenza M.N., Stein D.J., King D.L., Hodgins D.C., Saunders J.B., Griffiths M.D., Gjoneska B., Billieux J., Brand M. (2020). Preventing problematic internet use during the COVID-19 pandemic: Consensus guidance. Compr. Psychiatry.

[93] Kar S.K., Arafat S.M.Y., Sharma P., Dixit A., Marthoenis M., Kabir R. (2020). COVID-19 pandemic and addiction: Current problems and future concerns. Asian J. Psychiatr..

[94] Skoric M.M., Teo L.L., Neo R.L. (2009). Children and video games: Addiction, engagement, and scholastic achievement. Cyberpsycol. Behav..

[95] Pontes H.M., Griffiths M.D. (2019). A new era for gaming disorder research: Time to shift from consensus to consistency. Addict Behav..

[96] Holmes E.A., O’Connor R.C., Perry V.H., Tracey I., Wessely S., Arseneault L., Ballard C., Christensen H., Silver R.C., Everall I. (2020). Multidisciplinary research prior-ities for the COVID-19 pandemic: A call for action for mentalhealth science. Lancet Psychiatry.

[97] Pfefferbaum B., North C.S. (2020). Mental health and theCovid-19 pandemic. N. Engl. J. Med..

[98] Qiu J., Shen B., Zhao M., Wang Z., Xie B., Xu Y. (2020). A nationwide survey of psychological distress among Chinese people in the COVID-19 epidemic: Implications and policy recommendations. Gen. Psychiatry.

[99] Ko C.H., Yen J.Y. (2020). Impact of COVID-19 on gaming disorder: Monitoring and prevention. J. Behav. Addict..

[100] Snodgrass J.G., Lacy M.G., Dengah H.F., Eisenhauer S., Batchelder G., Cookson R.J. (2014). A vacation from your mind: Problematic online gaming is a stress response. Comput. Hum. Behav..

[101] Elhai J.D., Yang H., McKay D., Asmundson G.J. (2020). COVID-19 anxiety symptoms associated with problematic smartphone use severity in Chinese adults. J. Affect. Disord..

[102] King D.L., Gradisar M., Drummond A., Lovato N., Wessel J., Micic G., Douglas P., Delfabbro P. (2012). The impact of prolonged violent video-gaming on adolescent sleep: An experimental study. J. Sleep Res..

[103] Carter B., Rees P., Hale L., Bhattacharjee D., Paradkar S.M. (2016). Association between portable screen-based media device access or use in the sleep environment and sleep outcomes in children and adolescents. A systematic review and meta-analysis. JAMA Paediatr..

[104] Hale L., Emanuele E., James S. (2015). Recent Updates in the Social and Environmental Determinants of Sleep Health. Curr. Sleep Med. Rep..

[105] Fobian A.D., Avis K., Schwebel D.C. (2016). Impact of Media Use on Adolescents Sleep Efficiency. J. Dev. Behav. Pediatr..

[106] Michelle A., Gradisar M., Wright H., Lack L.C., Dohnt H., Carskadon M.A. (2011). Time for Bed: Parent-Set Bedtimes Associated with Improved Sleep and Daytime Functioning in Adolescents. Sleep Res. Soc..

[107] APA Resolution on Violent Videogames. https://www.apa.org/about/policy/resolution-violent-video-games.pdf.

[108] Hasan Y., Bègue L., Bushman B.J. (2013). Violent Video Games Stress People Out and Make Them More Aggressive. Aggress Behav..

[109] Mitrofan O., Paul M., Spencer N. (2009). Is aggression in children with behavioural and emotional difficulties associated with television viewing and video game playing? A systematic review. Child Care Health Dev..

[110] Greitemeyer T., Mügge D.O. (2014). Video Games Do Affect Social Outcomes: A Meta-Analytic Review of the Effects of Violent and Prosocial Video Game Play. Pers. Soc. Psychol. Bull..

[111] Smith P., Hawley P., Little T., Rodkin P. (2007). Aggression and Adaptation: The Bright Side to Bad Behavior.

[112] Ferguson C.J., Beaver K.M. (2009). Natural born killers: The genetic origins of extreme violence. Aggress. Violent Behav..

[113] Ferguson C.J., Dyck D. (2012). Paradigm change in aggression research: The time has come to retire the General Aggression Model. Aggress. Violent Behav..

[114] Kutner L., Olson C. (2009). Grand theft childhood: The surprising truth about violent video games and what parents can do. J. Commun..

[115] World Health Organization (2020). Violence, Health and Sustainable Development.

[116] Ibáñez V., Graña-Gómez J.L. (2018). Madurez psicosocial y comportamiento delictivo en menores infractores. Psicopatología Clínica Leg. Y Forense.

[117] Polman H., de Castro B.O., van Aken M.A.G. (2008). Experimental study of the differential effects of playing versus watching violent video games on children’s aggressive behavior. Aggress Behav..

[118] Zhang Q., Cao Y., Tian J.J. (2021). Effects of Violent Video Games on Aggressive Cognition and Aggressive Behavior. Cyberpsychol Behav. Soc. Netw..

[119] Robertson L.A., McAnally H.M., Hancox R.J. (2013). Childhood and adolescent television viewing and antisocial behavior in early adulthood. Pediatrics.

[120] Kuss D.J., Griffiths M.D. (2012). Adicción a los juegos en línea de los adolescentes. Educ. Y Salud.

[121] Chen V.H.H., Wilhelm C., Joeckel S. (2019). Relating video game exposure, sensation seeking, aggression, and socioeconomic factors to school performance. Behav. Inf. Technol..

[122] Adelantado-Renau M., Moliner-Urdiales D., Cavero-Redondo I., Beltrán-Valls M.R., Martínez-Vizcaíno V., Álvarez-Bueno C. (2019). Association Between Screen Media Use and Academic Performance Among Children and Adolescents. A Systematic Review and Meta-analysis. JAMA Pediatr..

[123] MacCann C., Jiang Y., Brown L.E., Double K.S., Bucich M., Minbashian A. (2020). Emotional Intelligence predicts academic performance: A meta-analysis. Psychol. Bull..

[124] Gros L., Debue N., Lete J., van de Leemput C. (2020). Video Game Addiction and Emotional States: Possible Confusion Between Pleasure and Happiness?. Front. Psychol..

[125] Miedzobrodzka E., Buczni J., Konijin E.A., Krabbendam L.C. (2021). Insensitive Players? A Relationship Between Violent Video Game Exposure and Recognition of Negative Emotions. Front. Psychol..

[126] Díaz R.L., Wong U., Hodgins D.C., Chiu C.G., Goghari V.M. (2016). Violent video game players and non-players differ on facial emotion recognition. Aggress Behav..

[127] Yen J.Y., Yen C.F., Chen C.S., Tang T.C., Ko C.H. (2009). The association between adult ADHD symptoms and internet addiction among college students: The gender difference. Cyberpsychol. Behav..

[128] González-Bueso V., Santamaría J.J., Fernández D., Merino L., Montero E., Ribas J. (2018). Association between internet gaming disorder or pathological video-game use and comorbid psychopathology: A comprehensive review. Int. J. Environ. Res. Public Health.

[129] Ko C.H., Yen J.Y., Chen C.S., Yeh Y.C., Yen C.F. (2009). Predictive values of psychiatric symptoms for internet addiction in adolescents: A 2-year prospective study. Arch. Pediatr. Adolesc. Med..

[130] Diamond A. (2005). Attention-deficit disorder (attention-deficit/ hyperactivity disorder without hyperactivity): A neurobiologically and behaviorally distinct disorder from attention-deficit/hyperactivity disorder (with hyperactivity). Dev. Psychopathol..

[131] Berloffa S., Salvati A., D’Acunto G., Fantozzi P., Inguaggiato E., Lenzi F., Milone A., Muratori P., Pfanner C., Ricci F. (2022). Internet Gaming Disorder in Children and Adolescents with Attention Deficit Hyperactivity Disorder. Children.

[132] Cabelguen C., Rocher B., Leboucher J., Schreck B., Challet-Bouju G., Hardouin J.B., Grall-Bronnec M. (2021). Attention deficit hyperactivity disorder and Gaming Disorder: Frequency and associate factors in a clinical sample of patients with Gaming Disorder. J. Behav. Addict..

[133] Wang B.-Q., Yao N.-Q., Zhou X., Lio J., Lv Z.-T. (2017). The association between attention deficit/hyperactivity disorder and internet addiction: A systematic review and meta-analysis. BMC Psychiatry.

[134] Weiss M.D., Baer S., Allan B.A., Saran K., Schibuk H. (2011). The screens culture: Impact on ADHD. Atten. Defic. Hyperact. Disord.

[135] Grusser S.M., Thalemann R., Albrecht U., Thalemann C.N. (2005). Excessive computer usage in adolescents—Results of a psychometric evaluation. Wien Klin Wochenschr.

[136] Entertainment Software Rating Board. https://www.esrb.org/tools-for-parents/parental-controls/.

[137] Ministerio de Educación, Cultura y Deporte Monográfico: Herramientas de control del aula—TcosMonitor. http://recursostic.educacion.es/observatorio/web/eu/equipamiento-tecnologico/aulas-digitales/938-monografico-herramientas-de-control-del-aula?start=3.

[138] Teng Z., Nie Q., Guo C., Zhang Q., Liu Y., Bushman B.J. (2019). A longitudinal study of link between exposure to violent video games and aggression in Chinese adolescents: The mediating role of moral disengagement. Dev. Psychol..

