# Supplementary material for: Internet and Video Games: Causes of Behavioral Disorders in Children and Teenagers
Source: Children (Basel). 2022 Dec 31;10(1):86. doi: 10.3390/children10010086 (PMC9856521; doi:10.3390/children10010086)
Supplement: Supplementary file 1 [file children-10-00086-s001.zip › children-2101584-supplementary.pdf]

Author(s)	Objective(s)	Instruments	Sample	Age	Country	Results
Yoo et al., 2004 [76]	To investigate the relationship between Internet addiction and attention deficit-hyperactivity/ impulsivity symptoms	- DuPaul's attention deficit hyperactivity disorder rating scale (K-ARS) (Korean version)<br>- Child Behavior Checklists (CBCL) (Korean version)<br>- Conner's scale (Korean version)<br>- Young's Internet Addiction test (IAT).	535 elementary school students	9-13 years old (M=11.1; SD=1)	South Korea	- Men had higher levels of Internet addiction than women ($p<.01$)<br>- Internet addiction was positively and significantly related to inattention ($p<.001$), hyperactivity-impulsivity ($p<.001$), and total attention deficit hyperactivity disorder ($p<.01$).<br>- In the Internet addiction group, playing games was the chosen option when they used Internet (instead of chatting, news…). In fact, the rate of games as the main usage is higher in the Internet addiction group ($p<.01$).<br>- The ADHD group had significantly higher addition that the non-ADHD group ($p<.001$).
Sheese and Graziano, 2005 [77]	To investigate how violent video games affect cooperative or competitive behavior	- Violent and nonviolent versión of Doom (game)<br>- PD-ALT decision matrix	548 students	-	United States	- Violent video game users are significantly more like to choose to defect than nonviolent video game users ($p=.03$).<br>- Playing violent video games fosters competitive behaviors and has negative effects on social behaviors too. However, playing violent video games did not cause participants to anticipate less cooperation or prosocial behavior from their parents.
Kirsh and Mounts, 2007 [78]	To investigate the effect of violent video games on the positive and negative emotions	- Video game rating form<br>- The NimStim Face Stimulus Set<br>- Dynamic emotion identification task (DEIT)	197 students	17-23 years old	New York, United States	- Females found video games more frustrating ($p<.01$) and felt less excitement ($p<.05$) than men.<br>- Students rated the violent video game as more humorous than the nonviolent ($p<.001$).

						- Boys enjoyed playing videogames more than girls (p<.001), and they preferred violent video game (p<.001).<br>- Females found nonviolent video games as the most difficult game (p<.02).<br>- Students who played violent video games presented a significantly reduced happy-face advantage (p<.05).
Wei, 2007 [79]	To investigate the influence of violent video games on adolescents' attitudes, empathy, and aggressive behavior.	- Initial questionnaire about playing video games<br>- Questionnaire about exposure to violent video games<br>- Attitudes toward violence scale.<br>- The empathic concern subscale<br>- The aggression scale	312 people	11-22 years old (M=16.91; SD=2.43)	China	- The average time that people spent using computers was 4.06h/day.<br>- Adolescents who spent more time playing violent video games would be more pro-violent and less concerned about others. There was a significant and positive correlation between exposure to violent video games and attitudes to violence (p<.01) and a significant but negative correlation between playing violent video games and empathy (p<.001).<br>- The relationship between exposure to violent video games and aggression was positive and significant (p<.01).<br>- Men spent more time playing violent video games (p<.001).
Sharif et al, 2010 [80]	To investigate the impact of using visual media on teenagers' school achievement.	- Interview via telephone about behavior, parenting style, and adolescents' disposition<br>- Questions for both, children and their parents about academic performance	4533 teenagers	10-14 years old (M=12)	United States	- Academic performance is negatively and significantly correlated to having a TV in bedrooms (p<.0001), hours viewing TV (p<.0001), and hours playing video games (p<.0001).<br>Teenagers with average/below average academic performance had a television in the bedroom (68%), spent more than two

						hours watching TV (37%), and more than one-hour playing video games (39%).<br>- Academic performance had a negative and significative relationship with the number of PG-13 movies and R-movies watched (p<.0001).<br>- School problem behavior is positively and significatively correlated to hours viewing TV, hours playing video games, and having a TV in bedrooms (p<.0001).<br>- Self-control is negatively and significantly correlated to hours viewing TV (p<.0001), and hours playing video games (p<.0001).
Rodríguez and Sandoval, 2011 [81]	To investigate the relationship between videogames use and memory, attention, academic performance, and behavioral problems	- Preliminary Questionnaire for Parents of Family<br>- Visual and hearing attention test<br>- Child Neuropsychological Test<br>- Child Behavior Checklist	99 children	8-13 years old	Colombia	- No significant differences were found between videogames use and academic performance.<br>- Hypothesis related to memory, and visual and hearing attention were not accepted because results were not enough. However, through these variables, significant differences between children were found in the influence of daily use of computers.
Wolfe et al., 2014 [82]	To investigate the relationship between the use of videogames before getting asleep and the sleep duration, working memory, and sustained attention	- Bioshock Infinitive game for PlayStation 3<br>- MicroMini Motion logger<br>- Computerized version of "Operation Span Task"<br>- Psychomotor Vigilante Task<br>- Video cameras at the sleep laboratory	21 healthy teenagers who used to sleep well	15-20 (M=17.6 years, SD=1.8)	Finder, Australia	- There is a significant and negative correlation between gaming time and sleep duration (p<.001) and between gaming time and sustained attention (p<.01). There is not any significant correlation with working memory.<br>- There is a significant and positive correlation between sleep duration and sustained attention.

						- Older teenagers spent less time videogaming (p<.05) and spent more time sleeping (p<.05).<br>- The relationship between video games and sustained attention was mediated by sleep duration.
Yousef et al., 2014 [83]	To investigate the relationship between the time spent on TV and videogame use with behavioral problems, sociodemographic characteristics, medical history, and diverse psychosocial factors	- Semi-structured questionnaire.<br>-Child Behavior Checklist (CBCL) (Arabic and parent version)	197 children	5-15 years (M=8.7; SD=2.1)	United Arab Emirates	- Younger in birth order or children that had fewer siblings, usually spent more than 2 hours per day with TV or video game.<br>- Children who spent more than 2 hours per day watching TV or playing video games, had higher scores on withdrawn (p=.001), attention (p=.002) and social (p=0.3) problems, and aggressive (p=.018) and delinquent (p=.023) behavior.<br>- There were not significant differences in anxiety, depression, thought problems, and somatic complaints between children who used the screen for more than 2 hours and those who used it less. (p>.05).
Weinstein, et al., 2015 [84]	To explore the correlation between ADHD and internet addiction.	- Young's Internet Addiction Test (IAT)	100 male children; 50 with attention deficit and hyperactivity disorder	13-15 years old (M=13.9; SD=0.72)	Israel	- Children with ADHD had higher levels of internet addiction than children without ADHD diagnosis, with significant differences (p<.01).<br>- ADHD group spent more hours using the Internet or video games (p<.05).<br>- ADHD group went to sleep later, most of them after 12 pm<br>- Results showed a relationship between ADHD, sleep disorder, and internet/video games addiction.

Cheung et al., 2017 [85]	To investigate whether the frequency of the digital screen is related to sleep	- Online survey for parents about their children's media use and sleep patterns	715 babies	6-36 months	United Kingdom	- A significant and positive association between digital screen use and sleep time during the day ($p<.05$) can be observed. Also, sleep onset ($p<.001$) was positively related to digital screen use. On the contrary, the relation between the digital screen and sleep time at night is negative ($p<.001$).<br>- There was not any significant correlation between digital screen use and the frequency of night awakenings.<br>- TV exposure and its duration were negatively related to sleep during the day ($p=.02$).<br>- An increase in digital screen use was associated with a decrease in the overall amount of sleep.
Yilmaz et al., 2018 [86]	To explore the impact of playing video games on the classmates and teachers of players	- Peer Association Test<br>- Student-Teacher Association Test<br>- Video games patterns Test<br>- Interviews	18 students and 2 teachers	9-10 years old (M=9.4)	Turkey	- Hardcore players showed lower academic performance in general. Some classmates affirmed that they helped hardcore players by doing their homework.<br>- English teacher mentioned that playing video games helped to learn English.<br>- Girls found difficult to communicate with hardcore gamers (HG) because of the topic of conversation. Teachers had poor communication with HG. Communication between HG is solid.<br>- Following teachers' and classmates' answers, it is identified that HG had affective, verbal (mockery, ridicule), self-

						control, and behavioral (intimidation and physical aggression) problems.<br>- Teachers affirmed that HG had attention problems and was more aggressive.
Horowitz-Kraus and Hutton, 2018 [87]	To investigate whether the time children spent with screen media or reading have effects on brain areas	- Test of Nonverbal Intelligence<br>- Vocabulary Task<br>- Woodcock-Johnson Tests of Cognitive Abilities<br>- Conners Parent Short-form Assessment<br>- StimQ-P instrument<br>- CONN functional connectivity toolbox in SPM8	19 children.	8-12 years old (M=9.99; SD=0.84)	United States	- 3.98 hours is the average that the sample spent in front of a screen.<br>- The number of hours that students spent reading were positively correlated to higher connectivity between regions related to visual word and language, visual association, and cognitive-control ($p<.05$).<br>- Functional connectivity between visual word form area, cognitive control, and language regions, was negatively correlated to screen-time hours.
Chindamo et al., 2019 [88]	To investigate the relationship between new screen-based electronic devices and sleep disorders.	- A Questionnaire with demographic data; breastfeeding and kindergarten attendance information; child's sleeping behavior; video game, screen-based media, and television use; bedtime routines; and questions about restlessness and sociability.	1.117 toddlers	12-23 months (M=25.3 months, SD=0.37)	Italy	- Frequency of video game playing had a significative impact on sleep onset latency ($p<.005$), increasing the time to get sleep, but the differences in total sleep time are not significative although it was reduced.<br>- Daily Tablet or mobile use increased reduced the total sleep time ($p<.05$) and increased the sleep onset latency ($p<.05$).
Cabré-Riera et al., 2019 [89]	To explore the relationship between telecommunication and screen device	- Self-reported questionnaires<br>- Mobile Phone Problematic Use Scale	226 adolescents for subjective	17-18 years old	Spain	- Phone calls and habitual mobile phone use were associated with worse quality of sleep.<br>- An increase in tablet use is negatively associated with sleep efficiency. Moreover,

	use, with sleep measures	- ActiGraph wGT3X-BT	measures and 110 adolescents for objective measures			that use increased minutes of wake time after sleep onset.
Salih et al., 2020 [90]	To investigate the negative effect of video games on children and teenagers	- Pre-structured questionnaire about sociodemographic data and use of videogames	303 children	4-17 years old	Saudi Arabia	- 99.3% of the sample played video games. 99% had their own devices. More than 50% used it during school days.<br>- 59,7% preferred playing video games rather than studying.<br>- Violence and aggressive behavior were associated with video games in 25% of the sample, and 37% affirmed that they ate a lot while playing video games.<br>- Videogames predisposed to lack of attention (23%), hyperactivity (19%), lower academic performance (32%), sleep disorders (45%), and addictive effects (5.9%).
Teng et al., 2021 [91]	To investigate the impact of COVID-19 on the relationship between internet gaming disorder, depressive symptoms, anxiety symptoms, and video game use.	- Demographic test<br>- Previously reported a method to assess video game use [146]<br>- The Internet Gaming Disorder Scale-Short Form (IGDS9-SF)<br>- Perceived COVID-19 impacts assessment<br>- Center for Epidemiologic Studies	1.778 children and teenagers	-	China	- Video game use ($p<.001$), increased exponentially after the COVID-19 pandemic in both, children, and adolescents.<br>- Internet gaming disorders and anxiety symptoms also increase in adolescents with significant differences ($p<.05$) after the pandemic, but without significant differences in children ($p>.05$).<br>- Depressive symptoms have no significant differences in any group.<br>- Video game use (T1) predicted internet gaming disorder (T1) and anxiety

		Depression Scale (CES-D) (Chinese version)<br>- The State-Trait Anxiety Inventory (STAI) (Chinese version)				symptoms (T1) with significant correlation. However, it has not a significant correlation with anxiety symptoms (T2).<br>- In T2, anxiety symptoms and video game use are not correlated, but they are with internet gaming disorders (p<.001).
Nosetti et al., 2021 [92]	To explore the association between pre-sleep habits, use of technology, and sleep.	- School Sleep Habits Survey (SSHS) (Italian version)<br>- Self-report test about the use of technology	972 teenagers divided into five groups:<br>- Group I (13-14 years old)<br>- Group II (15)<br>- Group III (16)<br>- Group IV (17)<br>- Group V (18-19 years old).	14-19 years old (M=15.7; SD=1.48)	Lombardy, Italy	- There were differences in sleep patterns between groups. Grupo III had frequent nocturnal awakenings (24,1%) and the highest percentage of bad sleepers (26,6%). Group IV presented difficulties in waking up in the morning (70,1%). Group V had difficulties falling asleep (42.7%) and the highest percentage of insufficient sleep (40.4%).<br>- There was a negative correlation between the use of mobile phones (p=.03) or the Internet (p<.01) as pre-sleep habits and sleep time.<br>- Using of mobile phone (p=.003) or Internet (p=.008), playing videogames (p=.009), and listening to music (p=.001) as pre-sleep habits had a positive correlation to late bedtime.
